# Effect of Nanostructuring on the Thermoelectric Properties of β-FeSi_2_

**DOI:** 10.3390/nano11112852

**Published:** 2021-10-26

**Authors:** Linda Abbassi, David Mesguich, David Berthebaud, Sylvain Le Tonquesse, Bhuvanesh Srinivasan, Takao Mori, Loïc Coulomb, Geoffroy Chevallier, Claude Estournès, Emmanuel Flahaut, Romain Viennois, Mickaël Beaudhuin

**Affiliations:** 1 ICGM, Univ. Montpellier, CNRS, ENSCM, Montpellier, France; loic.coulomb@umontpellier.fr (L.C.); romain.viennois@umontpellier.fr (R.V.); 2 CIRIMAT, CNRS, Université de Toulouse, 118 Route de Narbonne, CEDEX 9, 31062 Toulouse, France; mesguich@chimie.ups-tlse.fr (D.M.); chevallier@chimie.ups-tlse.fr (G.C.); estourne@chimie.ups-tlse.fr (C.E.); flahaut@chimie.ups-tlse.fr (E.F.); 3 CNRS-Saint Gobain-NIMS, IRL 3629, Laboratory for Innovative Key Materials and Structures (LINK), National Institute for Materials Science (NIMS), Tsukuba 305-0044, Japan; david.berthebaud@cnrs.fr (D.B.); LETONQUESSE.Sylvain@nims.go.jp (S.L.T.); s.bhuvanesh5@gmail.com (B.S.); 4 MANA, National Institute for Materials Science (NIMS), 1-1-1 Namiki, Tsukuba 305-0044, Japan; MORI.Takao@nims.go.jp; 5 Plateforme Nationale CNRS de Frittage Flash, PNF2, MHT, Université Paul-Sabatier, 118 Route de Narbonne, CEDEX 9, 31062 Toulouse, France

**Keywords:** powder technology, sintering, nanocrystalline materials, thermal properties, semiconductors, energy storage and conversion

## Abstract

Nanostructured β-FeSi_2_ and β-Fe_0.95_Co_0.05_Si_2_ specimens with a relative density of up to 95% were synthesized by combining a top-down approach and spark plasma sintering. The thermoelectric properties of a 50 nm crystallite size β-FeSi_2_ sample were compared to those of an annealed one, and for the former a strong decrease in lattice thermal conductivity and an upshift of the maximum Seebeck’s coefficient were shown, resulting in an improvement of the figure of merit by a factor of 1.7 at 670 K. For β-Fe_0.95_Co_0.05_Si_2_, one observes that the figure of merit is increased by a factor of 1.2 at 723 K between long time annealed and nanostructured samples mainly due to an increase in the phonon scattering and an increase in the point defects. This results in both a decrease in the thermal conductivity to 3.95 W/mK at 330 K and an increase in the power factor to 0.63 mW/mK^2^ at 723 K.

## 1. Introduction

Global warming and the energy crisis have increased the interest in renewable and green energy sources. Among them, thermoelectricity, which enables the conversion of the loss of heat into electricity, benefits from recent advances thanks to the finding of new families of materials and to the development of new concepts such as multi-scale materials. A thermoelectric material can be characterized by its dimensionless figure of merit ZT = α^2^σT/λ_Tot_, where α is the Seebeck’s coefficient (V/K), σ is the electrical conductivity (S/m) and λ_Tot_ is the total thermal conductivity (W/mK) combining lattice λ_L_ and charge carrier contribution λ_e_ [1]. The efficiency of a thermoelectric module is proportional to the figure of merit, consequently, it is expected to reach a high ZT to obtain high performances. However, the efficiency is still limited for the thermogeneration of electricity compared to electricity produced by turbines [2], and most of the new thermoelectric materials, as well as the conventional materials, are made of rare, expensive and toxic elements such as chalcogen or pnictogen atoms or have stability problems [3,4,5]. To overwhelm these last problems, silicide intermetallics such as Mg_2_Si_1-x_Sn_x_, higher manganese silicides or β-FeSi_2_ were developed [6]. This last material has been investigated for several decades for its thermoelectric properties [7]. It crystallizes in the orthorhombic structure *Cmce* (Space group 64) [8] and is a semiconductor that can be both *n* or *p* type with an indirect bandgap around 0.8 eV [9,10,11]. Its power factor $\alpha^{2}\sigma$ is negligible when it is pure and its total thermal conductivity λ _Tot_ ranges from 10 to 18 W/mK at 300 K [11,12]. After alloying with Al or Co, $\alpha^{2}\sigma$ is similar to the best industrial thermoelectric (TE) materials [13] and its maximum figure of merit ZT reaches about 0.2 and 0.4, respectively [7] while it is 1 or greater for the best TE materials [14]. These low ZT values are mainly related to the high value of λ_Tot_ which is about 6.2 W/mK or 4.3 W/mK at 300 K for Al or Co alloying, respectively [7]. Recently, alloying with both Al and up to 20% of Os leads to an increase in the ZT of p-type β-FeSi_2_ up to 0.35 [15]. However, the use of Os is expensive and its oxide is very toxic and will limit its applications. Whereas the improvement of $\alpha^{2}\sigma$ can be explained by tuning of the charge carrier concentration, the decrease in λ_Tot_ can be explained by an increase in the phonon scattering by point defects and mass fluctuations. However, the lattice contribution to the thermal conductivity remains high and still represents >95% of λ_Tot_ [11]. Thus, there is a large potential for the reduction of thermal conductivity through nanostructuration. In the past, the large efficiency of ball milling for decreasing the crystallite size and so the thermal conductivity was shown. In such conditions, the ZT was increased by about 50% in the best usual thermoelectric materials such as alloys based on Bi_2_Te_3_, PbTe or Si-Ge [16,17,18]. An even larger effect was demonstrated in the case of n-doped silicon in which the thermal conductivity was divided by 15 and the ZT increased from 0.2 to 0.7 at 1273 K [19]. We also demonstrated a significant reduction of the thermal conductivity in several transition metal silicides through nanostructuration [20,21,22]. In this study, we investigated the effect of the nanostructuration on thermoelectric properties by combining a top-down approach coupled with Spark Plasma Sintering (SPS) on β-FeSi_2_ and one of the best Co-alloys β-Fe_0.95_Co_0.05_Si_2_.

## 2. Materials and Methods

Fe pieces (99.99%, Alfa Aesar, Karlsruhe, Germany), Si lumps (99.9999%, Alfa Aesar, Karlsruhe, Germany) and pre-alloyed CoSi with Co slug (99.95%, Alfa Aesar, Karlsruhe, Germany) were used as raw materials in stoichiometric ratio and melted in an arc-melting furnace under Ar atmosphere. For β-FeSi_2_ samples, the ingots were first sealed under vacuum in a quartz tube and annealed at 1123K for 50 h whereas β-Fe_0.95_Co_0.05_Si_2_ samples were used as-is. Both β-FeSi_2_ and β-Fe_0.95_Co_0.05_Si_2_ samples were crushed in an agate mortar and milled in a Fritsch ”Pulverisette 7” planetary micromill (Fritsch, Idar-oberstein, Germany). A silicon nitride container of 45 mL and five 15 mm-diameter balls were used as the milling media with a ball-to-powder mass ratio set to 10:1. The speeds of the supporting disc and the grinding bowl were 510 RPM and 1020 RPM, respectively, for all the experiments (ball acceleration ~80 m s^−2^). The grinding process was performed in a glovebox under an Ar atmosphere to avoid oxygen contamination. SPS technique was used to prepare dense pellets using a “Dr. sinter 632Lx” unit (Fuji Electronic Industrial Co., Ltd., Tsurugashima, Japan) at PNF2 (Toulouse, France). Tungsten carbide die with an inner diameter of 8 mm was used for those experiments with a graphite foil as a protective and lubricating layer between the samples and the die. Uniaxial pressure in the range 100–500 MPa and DC pulses were both delivered by tungsten carbide punches at both sides. A dwell time of 5 min was applied at a temperature between 873 K and 1073 K. For the Fe_0.95_Co_0.05_Si_2_ sample, the uniaxial pressure was 500 MPa and the sintering temperature was 873 K, the dwell time being still 5 min.

The samples were characterized by powder X-ray diffraction (Philips X’PERT, PANalytical B.V., Amsterdam, The Netherlands). Rietveld refinement with Fullprof or FAULTS software was used for structural analysis of each sample [23,24,25]. The microstructure was analyzed by HRTEM JEOL JEM 2100F (JEOL Ltd., Tokyo, Japan). $\lambda_{Tot}$ was derived from the thermal diffusivity *D* measured by the laser flash method from Netzsch (LFA 467 Hyperflash, Netzsch, Selb, Germany), the density d being determined from the Archimedes’ method and the specific heat $C_{p}$ by Pyroceram using the relationship $\lambda_{Tot}=DdC_{p}$. The electrical resistivity ρ=1/σ and Seebeck’s coefficient were measured using a homemade apparatus.

## 3. Results and Discussion

### 3.1. Structural Analysis

**Figure 1:**
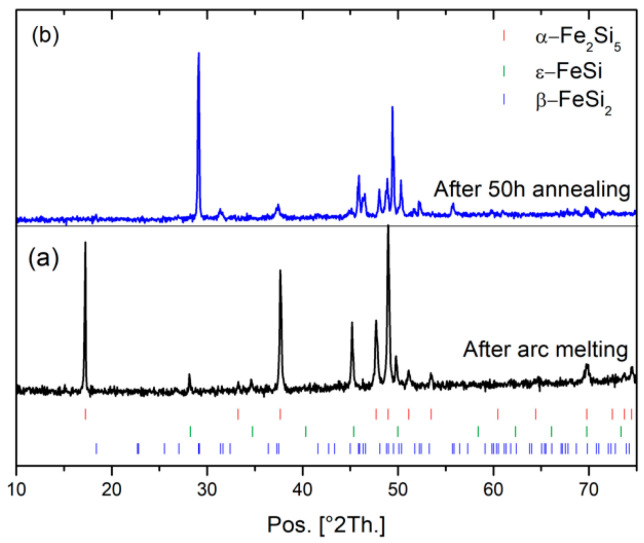
Diffraction pattern of β-FeSi_2_ samples after (**a**) arc melting and (**b**) 50 h annealing.

**Figure 2:**
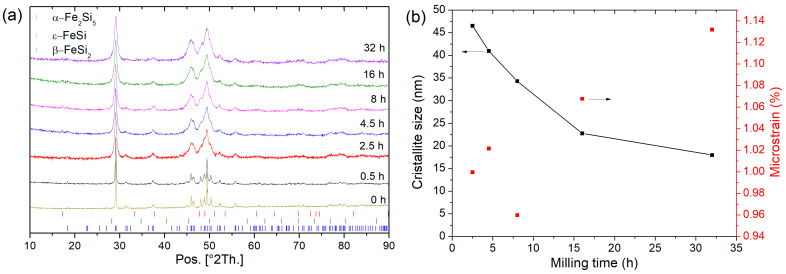
(**a**) Diffraction pattern of β-FeSi_2_ samples before and after mechanical milling (0.5 h to 32 h) and (**b**) crystallite size and microstrains. A guideline for the eyes (crystallite size) is shown.

**Figure 3:**
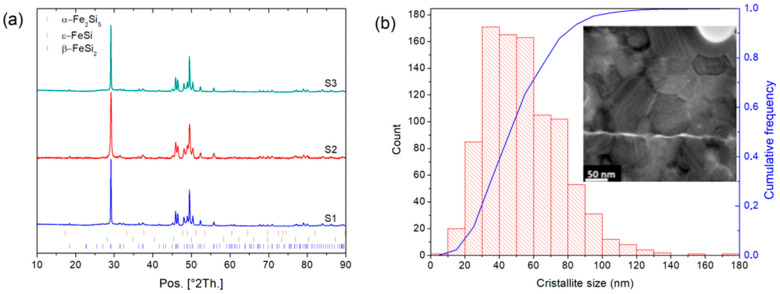
(**a**) X-ray Diffraction pattern of β-FeSi2 samples S1 to S3 obtained from the sintering of Fe-Si powder milled for 8 h. (**b**) Crystallite size distribution and HRTEM image of β-FeSi2 crystallite of sample S2 sintered at 500 MPa 873 K.

After arc melting of β-FeSi_2_ samples, the ingots are mainly composed of α-Fe_2_Si_5_ and ε-FeSi phases whereas after 50 h of annealing the Rietveld refinement shows that β-FeSi_2_ is obtained (Figure 1) with a unit cell parameter: a = 9.8767(7) Å, b = 7.8139(4) Å, and c = 7.8296(4) Å, in good agreement with the literature (a = 9.863 Å, b = 7.791 Å, and c = 7.833 Å) [8]. Increasing the milling time from 0.5 h to 32 h was investigated in Figure 2. The analysis of the XRD patterns shows a broadening of the peak feature with increasing the milling time. Rietveld refinement of these data shows a decrease in the crystallite size with increasing the milling time and an increase in the microstrains (Figure 2b). After 8 h of milling, the crystallite size reached ~34 nm and after 32 h of milling, it reached a plateau at ~17 nm. In the next step, the powder sintered by SPS was milled for 8 h, as it is a good compromise between small crystallite size and preparation time. In Table 1, the influence of the sintering conditions (temperature and pressure) on the relative density and the crystallite size of three β-FeSi_2_ samples (S1, S2 and S3) are summarized. For all samples, the β-FeSi_2_ phase is evidenced in the XRD patterns (Figure 3).

As expected, an increase in sintering temperature and/or pressure leads to an increase in the relative density. A minimum crystallite size of ~50 nm was obtained at 500 MPa and 873 K for the sample S2 as observed by HRTEM (Figure 3). We also note that an increase in the sintering temperature leads to an increase in the crystallite size, which is typical of grain coarsening. This is why for the sample S4 with five at. % Co on the Fe site, we used the same SPS conditions as for the sample S2, which is the best compromise between high density and small crystallite size. To investigate the effect of the crystallite size on the thermoelectric performances, a pellet S2_ann_ was sintered at 1073 K, 100 MPa for 5 min and annealed for 72 h at 1123 K. S2_ann_ exhibits a crystallite size above 200 nm and a relative density of 93.2%.

**Figure 4:**
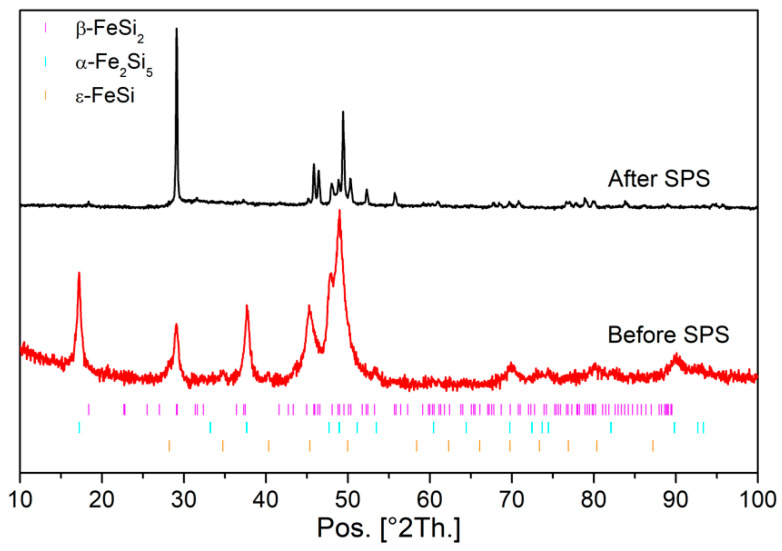
X-ray Diffraction pattern of β-Fe_0.95_Co_0.05_Si_2_ sample S4 obtained before and after the sintering of Fe-Co-Si powder milled for 10 h.

After arc melting and mechanical milling of the β-Fe_0.95_Co_0.05_Si_2_ sample, the powder is mainly composed of α-Fe_2_Si_5_ and ε-FeSi phases. After SPS of this powder, an in situ reaction occurs leading to β-Fe_0.95_Co_0.05_Si_2_ being obtained (Figure 4). The analysis of the diffraction pattern by Le Bail refinement shows that the unit cell parameters are a = 9.90614(51) Å, b = 7.81260(57) Å, and c = 7.82618(37) Å. The crystallite size reaches 110 nm whereas the microstrain reaches 0.14% slightly above that observed on pure β-FeSi_2_ samples under these sintering conditions. This could be explained by the in situ reaction which leads to stronger but still limited grain coarsening.

### 3.2. Lattice Dynamic of Bulk and Nanostructured β-FeSi_2_

In order to study the effect of the crystallite size on the phonons and their relaxation time, we performed Raman scattering experiments on *β-FeSi_2_* samples S2_ann._ and S2. *β-FeSi_2_* has an orthorhombic structure of space group *Cmce* with 24 atoms in the primitive cell and has therefore 69 optical modes and three acoustic modes. The group theory predicts for the Brillouin zone center vibrational modes the following decomposition in irreducible representations [26]:

Γ*_opt_* = 9*A_g_* ⊕ 9*B_1g_* ⊕ 9*B*_2*g*_ ⊕ 9*B*_3*g*_ ⊕ 9*B*_1*u*_ ⊕ 9*B*_2*u*_ ⊕ 7*B*_3*u*_ ⊕ 8*A_u_* and Γ*_ac_* = *B*_1*u*_ ⊕ *B*_2*u*_ ⊕ *B*_3*u*_

There are 36 Raman-active vibrational modes of *A_g_*, *B*_1*g*_, *B*_2*g*_ and *B*_3*g*_ symmetries and there are 25 Infrared-active vibrational modes of *B*_1*u*_, *B*_2*u*_ and *B*_3*u*_.

**Figure 5:**
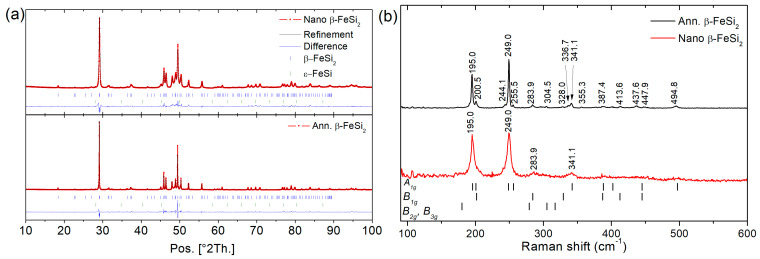
(**a**) Rietveld refinements of annealed β-FeSi_2_ (S2_ann._) and Nano β-FeSi_2_ (S2) using FAULTS. (**b**) Raman spectra of annealed S2_ann._ and nanostructured S2 β-FeSi_2_ sample (with assigned Raman mode [27]).

In Figure 5, we report the Raman spectra of S2_ann._ and S2 samples. The Raman spectrum extends from about 190 to 500 cm^−1^ and we found 16 Raman lines for the bulk S2_ann._ sample and four Raman lines in the nano S2 sample. Our results for the bulk S2_ann._ sample agree well with previous experimental results [27] and also with the first-principles calculations [26], as illustrated in Figure 5 in which we show the positions of the Raman-active modes assigned from polarized Raman scattering experiments on single crystal [27]. Comparing the Raman spectra of the samples S2_ann._ and S2, we do not see significant change of the positions of the four main lines but we observe a broadening of the full width at half maximum of all β-FeSi_2_ assigned modes from 2 cm^−1^ for the annealed sample S2_ann._ to 6 cm^−1^ for the nanostructured sample S2. The broadening of Raman bands could be explained by both the nanostructuration and the increase in defect contents in the crystal structure [28] induced during the mechanical milling. Moreover, this is characteristic of a lower relaxation time [29]. We consequently expect a lower thermal conductivity for nanostructured sample S2. Interestingly, one observes that the amount of stacking fault for samples with 50 nm crystallite size (S2) and 200 nm (S2_ann._) are respectively 12% and 19% whereas, in the literature [11], it was observed that it is about 11% for the sample with crystallite size between 0.5 and 1μm and 4 % for crystallite size between 1 to 10 microns. These results show that a decrease in the crystallite size is combined with an increase in the point defect concentration and stacking faults which could also participate in the phonon scattering.

### 3.3. Thermoelectric Properties

**Figure 6:**
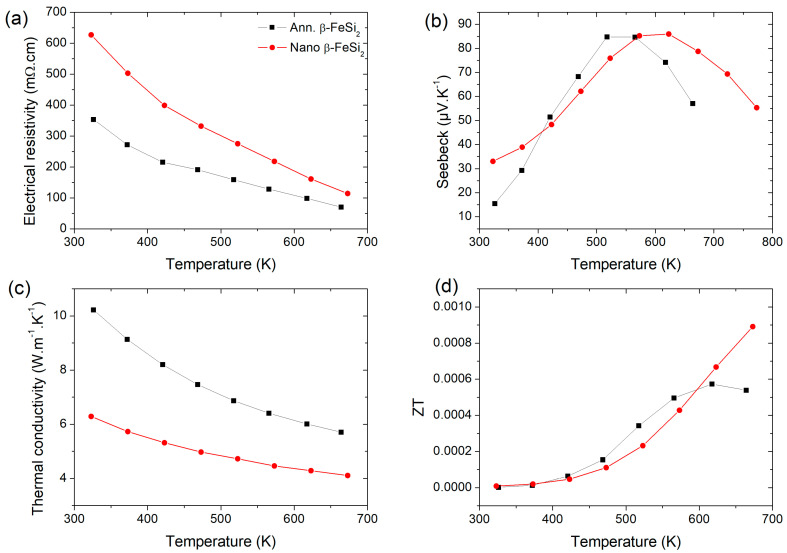
(**a**–**c**) Thermoelectric properties of annealed S2_ann_ and nanostructured S2 β-FeSi_2_ samples and their (**d**) figure of merit ZT.

**Figure 7:**
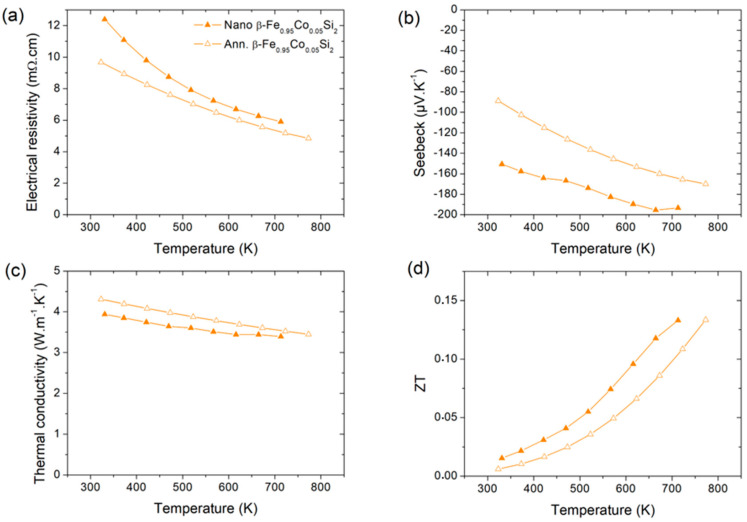
Thermoelectric properties of β-Fe_0.95_Co_0.05_Si_2_ (**a**) electrical resistivity, (**b**) Seebeck coefficient, (**c**) Thermal conductivity, (**d**) ZT.

Thermoelectric performances of S2 (after three temperature cycles to 673 K) and S2_ann._ β-FeSi_2_ samples are given in Figure 6. Figure 6a presents the decrease in the electrical resistivity of the annealed and nanostructured samples with temperature dependence which is typical of a semi-conductor. The electrical resistivity of the nanostructured sample is higher than the annealed sample due to an increase in electron scattering at the interfaces and by an increase in point defects due to mechanical milling [22]. The Seebeck’s coefficient is quite similar between both samples with a maximum of the thermopower that can be explained either by the change from low temperature extrinsic to high-temperature intrinsic conduction, which is characteristic of a change from heavily doped to lightly doped semiconductor [30] or by bipolar conduction effect as, e.g., in Bi_2_Te_3_ based alloys [31,32]. However, the energy bandgap of β-FeSi_2_ is around 0.8 eV [9,10,11], i.e., about four times larger than in these Bi_2_Te_3_ based alloys. This makes it very unlikely to have this effect in β-FeSi_2_. This is confirmed by the absence of an increase in the thermal conductivity at high temperatures, which is characteristic of bipolar conduction. It is interesting to note that there is an upshift in temperature of the Seebeck maximum when the sample is nanostructured. As both samples were prepared from the same powder, the only parameter that can be changed are defects in the crystal structure. Thus, we explain this upshift by the increase in defect content in the nanostructured sample that broadens the temperature range of the extrinsic regime. Similar weakly positive values of Seebeck’s coefficient were observed for β-FeSi_2_ by Tani and Kido [33] for samples containing traces of FeSi, i.e., with a deficit of Si probably due to Si vacancies which behave as acceptors, as conformed by DFT calculations [34,35]. The $\lambda_{Tot}$, which can be assimilated to the lattice contribution $\lambda_{L}$ (Figure S1), is decreased by a factor of 1.7 at 300 K and 1.5 at 670 K to reach a $\lambda_{Tot}$ similar to that of Co-doped samples, as reported earlier [11] (Figure S3) and as observed in Figure 7. This results in an improvement of the figure of merit at 670 K by a factor of 1.7 (Figure 6d) mainly due to the decrease in $\lambda_{Tot}$, as the power factor is similar for both samples (Figure S4).

The use of a multi-scale system combining nanostructuring and alloying could then enhance the thermoelectric performances and this can give a good opportunity to improve the performances of β-FeSi_2_ alloyed with Co. We then studied the case of the thermoelectric properties of the nanostructured β-Fe_0.95_Co_0.05_Si_2_ sample S4 (after six temperature cycles at 723 K) compared to a high temperature annealed sample (S4_ann._) for 72 h at 1123 K.

We did not observe any significant variation of the electrical resistivity of the S4 sample after six cycling to 723 K which means that the nanostructure is quite stable up to this temperature for viable application. Consequently, in Figure 7, the results presented correspond only to the last cycle, the details of the cycle are given in SI, Figure S5.

In Figure 7a, the doping of β-FeSi_2_ with Co has reduced the electrical resistivity by a factor of ~40 for both S4 and S4_ann._ samples compared to the nanostructured β-FeSi_2_ (S2) sample and, in Figure 7b, one observes that the Seebeck’s coefficient is also increased. One also observes that the increase in α and ρ is much stronger for nanostructured sample S4. This could be explained by the number of point defects such as p-type Si vacancies which are expected to be higher for nanostructured sample S4 than high temperature annealed one S4_ann_. Thus, the larger ρ and α should be due to lower charge carrier concentration. This leads to an increase in the power factor by a factor of 1.2 (See Figure S6).

The thermal conductivity is decreased by a factor ~1.5 for S4 and S4_ann._ compared to S2 leading to an improvement of the figure of merit by a factor ~14. It is slightly lower for the S4 sample and can be explained by the grain coarsening and the decrease in point defects in S4_ann._ samples which lead to an increase in the phonon relaxation rate [22].

Comparing with the bulk β-Fe_0.95_Co_0.05_Si_2_ samples in the literature, we find a ZT larger than Kim et al. [36] (ZT = 0.09 at 723 K) and similar to that of He et al. [37] (ZT = 0.15 at 723 K) but smaller than Tani et al. [38] (ZT = 0.19 at 723 K) and Hesse et al. [7] (ZT = 0.34 at 723 K). Note that this last result obtained in 1969 has never been reproduced. For all these bulk alloys, the thermal conductivity at 300 K was between 4.3 and 5 W/mK whereas in this study the nanostructured sample reaches 3.95 W/mK. We see therefore that a combination of both alloying and nanostructuring enable $\lambda_{Tot}$ to decrease again. We find an electrical resistivity larger than the previous works for both S4 and S4_ann._ samples which could be explained by the nanostructuration of the samples even after annealing. Such behavior has already been observed in the literature [39,40,41] and it is the main issue and challenge for nanostructured samples.

Now, if we compare these data to those from the literature [7,36,37,38,42,43], the gain of ZT is quite limited and shows compensation of the thermoelectric properties (lower λ and lower σ) as observed in previous studies [20,44] on other thermoelectric transition metal silicides. However, it is important to observe that a slight variation of the Co composition can lead to a strong variation of the thermoelectric performances [38] which means that it is important to compare samples from the same batch. Consequently, as observed in Figure 7, the nanostructuring of β-Fe_0.95_Co_0.05_Si_2_ is favorable to an improvement of the figure of merit and further optimization of the Co composition could permit to enhance significantly the figure of merit.

## 4. Conclusions

High-density nanostructured β-FeSi_2_ pellets can be successfully obtained from spark plasma sintering highly nanostructured powder. Grain coarsening is strongly limited by working in soft sintering conditions such as 873 K, 500 MPa for 5 min, at which the grain size is 50 nm. The thermoelectric performances of nanostructured undoped β-FeSi_2_ samples are above those of bulk samples due to a strong decrease in the lattice component of thermal conductivity and an upshift of the maximum Seebeck’s coefficient resulting in an enhancement of the figure of merit by a factor of 1.7 at 670 K. In the case of the nanostructured β-Fe_0.95_Co_0.05_Si_2_ sample, the electrical resistivity and electrical conductivity are strongly decreased compared to the nanostructured undoped β-FeSi_2_ samples but the electrical resistivity is higher than in the bulk β-Fe_0.95_Co_0.05_Si_2_ which is not fully compensated by the 10–20% reduction of the thermal conductivity in the nanostructured samples compared to the bulk samples, resulting in ZT = 0.14 at 723 K. This must be due to the smaller mean-free path of the phonons compared to the electrons in β-Fe_0.95_Co_0.05_Si_2_.

## Figures and Tables

**Table 1:** Influence of the sintering conditions (pressure and temperature) on the relative density and the crystallite size of β-FeSi_2_ and β-Fe_0.95_Co_0.05_Si_2_ for a dwell time of 5 min.

Sample	Composition	P (MPa)	T (K)	Relative Density (%)	Crystallite Size (nm)
S1	β-FeSi_2_	300	973	93.2	~185
S2	β-FeSi_2_	500	873	93.1	~50
S3	β-FeSi_2_	500	973	95.2	~320
S4	β-Fe_0.95_Co_0.05_Si_2_	500	873	93.7	~110

## Data Availability

Data is contained within the article or Supplementary Materials.

## Supplementary Materials

The following are available online at https://www.mdpi.com/article/10.3390/nano11112852/s1, Figure S1: Temperature dependence of the total thermal conductivity, $\lambda_{Tot}$, of annealed (S2_ann._) and nanostructured (S2) samples. The electronic component of the thermal conductivity, $\lambda_{e}$, was determined assuming that $L=L_{0}$, Figure S2: Temperature dependence of the total thermal conductivity, $\lambda_{Tot}$, of annealed (S4_ann._) and nanostructured (S4) samples. The electronic component of the thermal conductivity, λ_e, was determined assuming that $L=L_{0}$, Figure S3: Thermal conductivity of nanostructured S2 and annealed S2_ann._ samples compared to the literature data [S1], Figure S4: Temperature dependence of the power factor of annealed (S2ann.) and nanostructured (S2) samples, Figure S5: Temperature dependence of the electrical resistivity of S4 sample after six heating to 723 K, Figure S6: Temperature dependence of the power factor of annealed (S4_ann._) and nanostructured (S4) SPS samples. Table S1: Structural parameters and stacking faults probability (SF) of nanostructured β-FeSi_2_ (S2), annealed β-FeSi_2_ (S2_ann._) and β-FeSi_2_ from the literature data [S1] using FAULTS.
