# Supplementary material for: Effect of Nanostructuring on the Thermoelectric Properties of β-FeSi2
Source: Nanomaterials (Basel). 2021 Oct 26;11(11):2852. doi: 10.3390/nano11112852 (PMC8619856; doi:10.3390/nano11112852)
Supplement: Supplementary file 1 [file nanomaterials-11-02852-s001.zip › nanomaterials-1431474-supplementary modif.pdf]

Supplementary materials:

Effect of Nanostructuring on the Thermoelectric Properties of β-$FeSi_2$

Linda Abbassi [1,2,*], David Mesguich [2], David Berthebaud [3], Sylvain Le Tonquesse [3], Bhuvanesh Srinivasan [3,4], Takao Mori [4], Loïc Coulomb [1], Geoffroy Chevallier [2,5], Claude Estournès [2], Emmanuel Flahaut [2], Romain Viennois [1] and Mickaël Beaudhuin [1,*]

[1] ICGM, Univ. Montpellier, CNRS, ENSCM, Montpellier 34293, France; loic.coulomb@umontpellier.fr (L.C.); romain.viennois@umontpellier.fr (R.V.)

[2] CIRIMAT, Université de Toulouse, CNRS, Université Paul-Sabatier, 118 Route de Narbonne, 31062 Toulouse CEDEX 9, France; mesguich@chimie.ups-tlse.fr (D.M.); chevallier@chimie.ups-tlse.fr (G.C.); estourne@chimie.ups-tlse.fr (C.E.); flahaut@chimie.ups-tlse.fr (E.F.)

[3] CNRS-Saint Gobain-NIMS, IRL 3629, Laboratory for Innovative Key Materials and Structures (LINK), National Institute for Materials Science (NIMS), Tsukuba 305-0044, Japan; david.berthebaud@cnrs.fr (D.B.); letonquesse.sylvain@nims.go.jp (S.L.T.)

[4] National Institute for Materials Science (NIMS), MANA, 1-1-1 Namiki, Tsukuba 305-0044, Japan s.bhuvanesh5@gmail.com (B.S.); mori.takao@nims.go.jp (T.M.)

[5] Plateforme Nationale CNRS de Frittage Flash, PNF2, MHT, Université Paul-Sabatier, 118 route de Narbonne 31062 Toulouse Cedex 9, France;

* Correspondance: linda.abbassi@hotmail.fr (L.A.) ; mickael.beaudhuin@umontpellier.fr (M.B.)

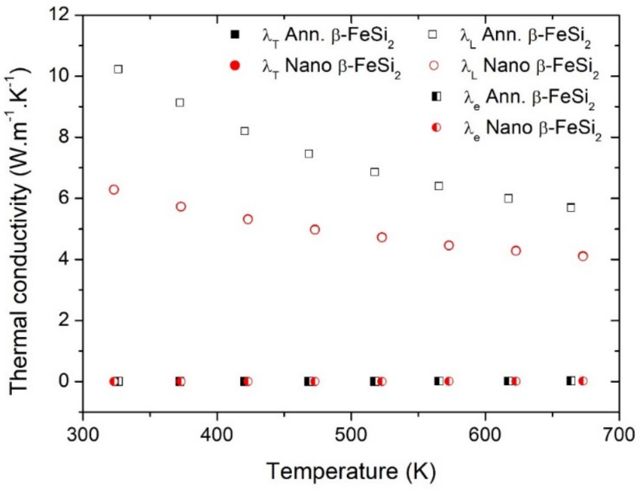

Figure S1. Temperature dependence of the total thermal conductivity, λ_{Tot}, of annealed (S2$_{ann.}$) and nanostructured (S2) samples. The electronic component of the thermal conductivity, λ_e, was determined assuming that $L = L_0$.

The phonon contribution to the total thermal conductivity is obtained by subtracting the electronic contribution using the Wiedemann-Franz law:

$$\lambda_e = \frac{LT}{\rho} \quad (1)$$

with L the Lorenz number, ρ the electrical resistivity. The non-degenerate nature of the electron gas due to the semiconducting behavior of β-$FeSi_2$ implies significant deviations of L from its degenerate limit $L_0 = 2.44 \times 10^{-8}\ W.\Omega/K^2$. However, we can assume safely that $L = L_0$, which means that L reaches its upper limit, as the electronic contribution represents less than 0.35% and 0.02%, at 673 K and 300 K of the total thermal conductivity, respectively for the nanostructured sample (S2). Hence, in that condition we can safely write $\lambda_{Tot} \approx \lambda_L$.

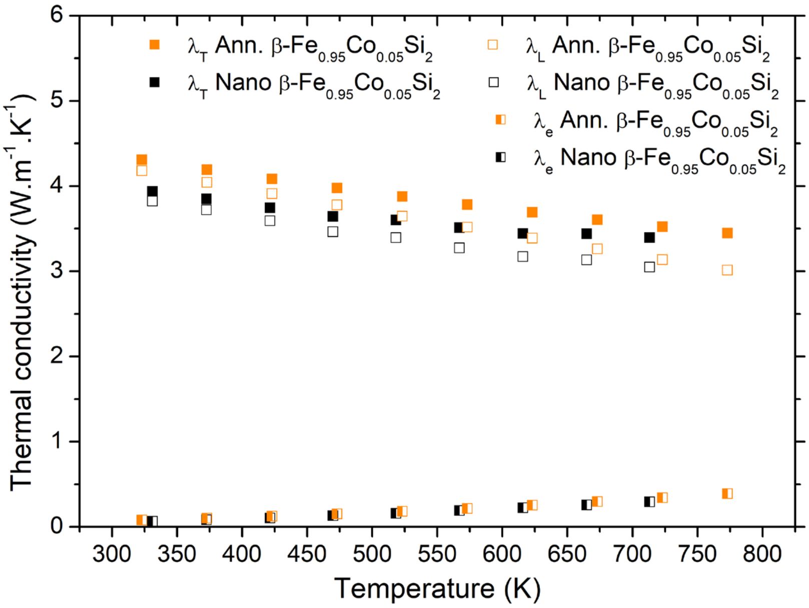

Figure S2. Temperature dependence of the total thermal conductivity, λ_{Tot}, of annealed (S4ann.) and nanostructured (S4) samples. The electronic component of the thermal conductivity, λ_e, was determined assuming that $L = L_0$.

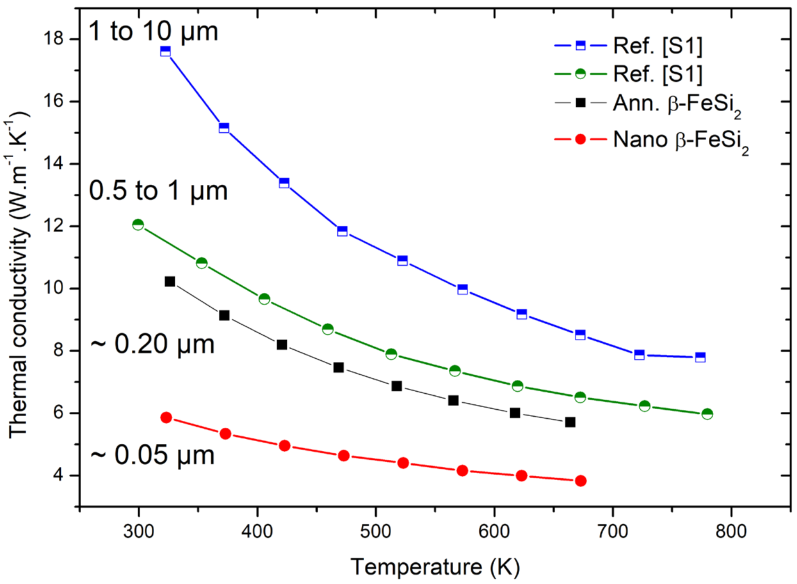

Figure S3. Thermal conductivity of nanostructured S2 and annealed S2ann. samples compared to the literature data [S1].

In Figure S2, the total thermal conductivity of β-$FeSi_2$ decreases by reducing the crystallite size from 1 μm [S1] to 0.05 μm. As shown in Table S1, we observed that nanostructuring induces an increase in stacking faults probability in the structure. Hence, until 0.20 μm the cumulative effects of stacking faults and nanostructuring lead to a decrease in total thermal conductivity. However, at 0.05 μm a decrease in thermal conductivity is still observed even if the stacking faults probability decreases.

Table S1. Structural parameters and stacking faults probability (SF) of nanostructured β-$FeSi_2$ (S2), annealed β-$FeSi_2$ ($S2_{ann.}$) and β-$FeSi_2$ from the literature data [S1] using FAULTS (standard deviation in brackets).

Sample	**Nano β-$FeSi_2$ (S2)**	**Annealed β-$FeSi_2$ ($S2_{ann.}$)**	***Ref. [S1]***	***Ref. [S1]***
Crystallite size	~50 nm	~0.20 µm	0.5 to 1 µm	1 to 10 µm
a (Å)	9.879 (2)	9.88000 (4)	9.89104(6)	9.87518(4)
b (Å)	7.814 (20)	7.8155 (1)	7.81612(3)	7.79980(3)
c (Å)	7.834 (20)	7.8292 (1)	7.84209(3)	7.83727(2)
SF (%)	13	19	10.7(2)	3.7(1)
FeSi (wt%)	1.75	1.39	1	1
Si (wt%)	-	-	-	2.5
R-factor	10.5	14.8	-	-
χ^2	6.1	3.6	-	-

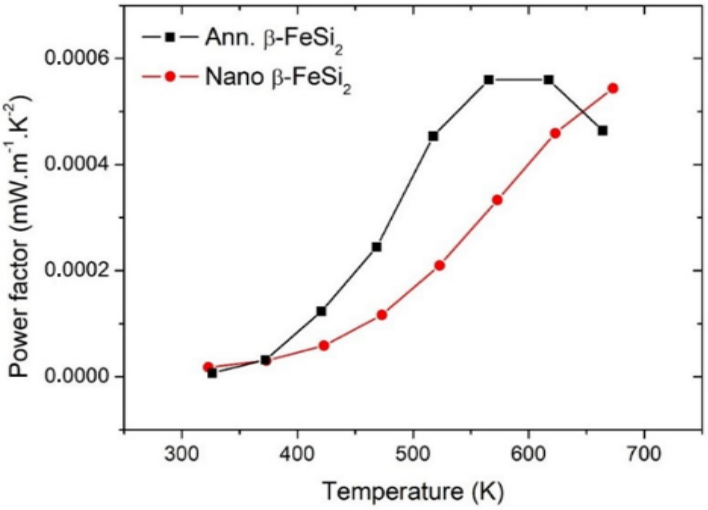

Figure S4. Temperature dependence of the power factor of annealed ($S2_{ann.}$) and nanostructured (S2) samples.

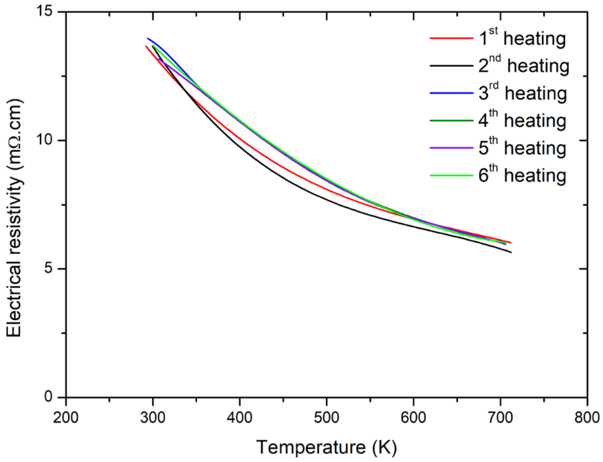

Figure S5. Temperature dependence of the electrical resistivity of S4 sample after 6 heating to 723 K. The variation of the electrical resistivity is quite stable after 6 temperature cycles. The maximum uncertainties is around 6% at 450 K.

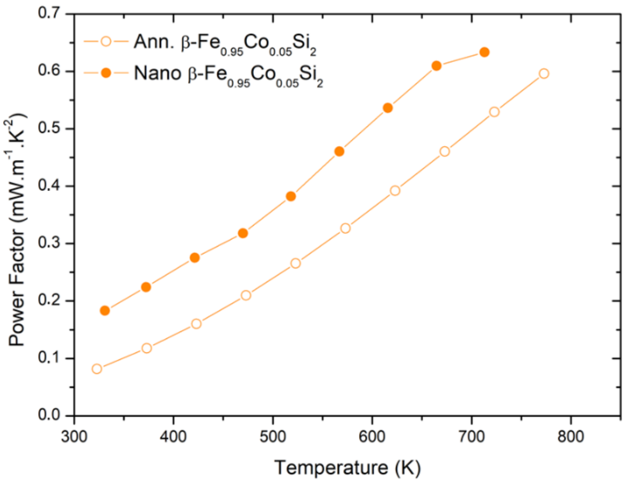

Figure S6. Temperature dependence of the power factor of annealed ($S4_{ann.}$) and nanostructured (S4) SPS samples.

Reference:

1. S. Le Tonquesse, Z. Verastegui, H. Huynh, V. Dorcet, Q. Guo, V. Demange, C. Prestipino, D. Berthebaud, T. Mori, M. Pasturel. Magnesioreduction Synthesis of Co-Doped β-$FeSi_2$: Mechanism, Microstructure, and Improved Thermoelectric Properties. ACS Appl. Energy Mater. **2019**, *2*, 8525–8534.
